# Molecular modeling, simulation and docking of Rv1250 protein from *Mycobacterium tuberculosis*

**DOI:** 10.3389/fbinf.2023.1125479

**Published:** 2023-04-12

**Authors:** Sumita Choudhary, Anup Kumar Kesavan, Vijay Juneja, Sheetal Thakur

**Affiliations:** ^1^ Department of Molecular Biology and Biochemistry, Guru Nanak Dev University, Amritsar, Punjab, India; ^2^ Department of Biotechnology and Microbiology, Kannur University, Dr. E. K. Janaki Ammal Campus, PalayadKannur, Kerala, India; ^3^ Eastern Regional Research Center, United States Department of Agriculture, Agricultural Research Service, Wyndmoor, PA, United States; ^4^ University Centre for Research & Development, Department of Biotechnology, Chandigarh University, Gharuan-Mohali, Punjab, India

**Keywords:** homology modeling, isoniazid, I-TASSER, molecular docking, molecular dynamics, tuberculosis

## Abstract

Computational prediction and protein structure modeling have come to the aid of various biological problems in determining the structure of proteins. These technologies have revolutionized the biological world of research, allowing scientists and researchers to gain insights into their biological questions and design experimental research much more efficiently. Pathogenic *Mycobacterium* spp. is known to stay alive within the macrophages of its host. *Mycobacterium tuberculosis* is an acid-fast bacterium that is the most common cause of tuberculosis and is considered to be the main cause of resistance of tuberculosis as a leading health issue. The genome of *Mycobacterium tuberculosis* contains more than 4,000 genes, of which the majority are of unknown function. An attempt has been made to computationally model and dock one of its proteins, Rv1250 (MTV006.22), which is considered as an apparent drug-transporter, integral membrane protein, and member of major facilitator superfamily (MFS). The most widely used techniques, i.e., homology modeling, molecular docking, and molecular dynamics (MD) simulation in the field of structural bioinformatics, have been used in the present work to study the behavior of Rv1250 protein from *M. tuberculosis*. The structure of unknown TB protein, i.e., Rv1250 was retrived using homology modeling with the help of I-TASSER server. Further, one of the sites responsible for infection was identified and docking was done by using the specific Isoniazid ligand which is an inhibitor of this protein. Finally, the stability of protein model and analysis of stable and static interaction between protein and ligand molecular dynamic simulation was performed at 100 ns The designing of novel Rv1250 enzyme inhibitors is likely achievable with the use of proposed predicted model, which could be helpful in preventing the pathogenesis caused by *M. tuberculosis*. Finally, the MD simulation was done to evaluate the stability of the ligand for the specific protein.

## 1 Introduction

*M. tuberculosis* is a bacteria of pathogenic (Acid-fast, Gram-positive) species from the Mycobacteriaceae family that is known to be a causative agent of tuberculosis (Van der Zanden et al., 2002). It is evident from global tuberculosis report (2022) of World Health Organization (WHO) that tuberculosis (TB) is the major source of mortality among humans in spite of a curable contagious ailment. As per the report, 1.6 million people died from TB, including 1,87,000 people with human immunodeficiency virus (HIV) in year 2021 and 10.6 million people fell ill due to TB, globally. The propagation of extensively drug-resistant tuberculosis (XDR-TB), multi drug-resistant tuberculosis (MDR-TB) and totally drug-resistant tuberculosis (TDR-TB)-drug defiant strains of *M. tuberculosis* is considered to be the main cause of renaissance of tuberculosis as a leading health issue. Hence, it is required to have detailed research and advancement of certain novel and improved medicine for the treatment of tuberculosis (Basso et al., 2005).

The permeable obstruction present in the cell wall is the major feature due to which *M. tuberculosis* construct the intrinsic resistance against a variety of antimicrobials and the compounds causing lethality to cells. The movement of hydrophobic molecules into the cells is restricted due to the presence of outer arabinogalactan and peptidoglycan coverings, whereas the passage of both hydrophilic as well as hydrophobic particles is restricted by the mycolic acid present in the cell (Josa et al., 2008). Certain aqua-phobic antibiotics, for example, fluoroquinolone and rifampicin, have the ability to go into the bacterial cells by means of diffusion into the hydrophobic coating. Conversely, the nutrients and antibiotics those are hydrophilic in nature and not able to enter the cells via diffusion through the cell wall, get entry into the cell via porin. Porins are the beta-barrel proteins that act as the pore on the external membrane of bacteria for the passage of solutions, including hydrophilic ones. Due to the distinctive structure of bacterial cells, antibiotic target gene mutations are known to be the primary cause of therapeutic drug resistance (Bhutani et al., 2015; Armando et al., 2018).

Hence, in order to design a model for prevention of this disease, docking of this protein is done by ligand Isoniazid/Isonicotinic acid hydrazide (INH) which is considered as the foundation of tuberculosis chemotherapy and is used as the preventive treatment of tuberculosis. The catalase-peroxidase enzyme (KatG) is used to activate the INH, a pro-drug. Then there is a reaction of resulting active species with NAD+ (Nicotinamide adenine dinucleotide) to develop an INH-NAD adduct, which impedes the enoyl acyl carrier protein reductase (InhA), causing the inhibition of mycolic acid biosynthesis and hence, the mycobacterial cell death (Kumar & Jena, 2014; Abid et al., 2017). Mutation in KatG gene, a virulence determinant and activator of prodrug INH, is the chief mechanism of resistance of INH. S315T is a precise KatG variant which is located in approximately 94% of INH-resistant experimental isolates. Transmutation in the promoter region of inhA (c-15t) is followed as a secondary method of INH resistance, leading to the over-expression of inhA and hence the drug titration. There are several other techniques of INH resistance followed in different models which take account of redox change, activator expression changes of drugs, drug inactivation, and efflux pump activation (Josa et al., 2008; Bhutani et al., 2015).

Molecular docking is a computational technique that helps in prediction and conformation of a receptor-ligand complex, where ligand acts as a small molecule and the receptor can be a nucleic acid or protein. This dock system may produce several sites for the ligand in the receptor binding compartment (Basso et al., 2005). Although there are several *in-vitro* drug discovery approaches available for the evaluation of numerous components against a pre-identified target, the process remains expensive and lengthy. Consequently, molecular docking is an alternative approach that provides every possible location of the ligand followed by the selection of the best one.

*M. tuberculosis* Rv1250 protein is a potential molecular target that can be used in the development and design of new drugs to combat tuberculosis (Telenti et al., 1993). Earlier, the effect of anti-tuberculosis drugs therapy on mRNA efflux pump gene expression of Rv1250 in *M. tuberculosis* collected from TB patients was studied (Umar et al., 2019). The major object of our study is to design the novel Rv1250 enzyme inhibitors by using the predicted molecular docking model which could be helpful in determining the best position for ligand binding and hence for the invention of new drug to preventing the pathogenesis caused by *M. tuberculosis*. As the TB is second infectious leading destroyer after COVID-19 (Global Tuberculosis Report, WHO, 2022), the *in silico* target analysis for *M. tuberculosis* could help in new drug discovery.

## 2 Materials and methods

### 2.1 Receptor preparation and structure prediction

The amino acid sequence of a particular target protein, Rv1250, was retrieved from National Center for Biotechnology Information, i.e., NCBI (http://www.ncbi.nlm.nih.gov/) which is a part of the United States National Library of Medicine (NLM), a branch of the National Institutes of Health (NIH). It addresses a sequence of databases related to biomedicine and biotechnology and a significant source for bioinformatics tools and services. For the structure identification, SWISS-MODEL and Phyre2 servers were used which presented only 17.21% of the total predicted structure (Supplementary Figure S1, S2) (template: 6G9X) (Schwede et al., 2003; Kelley et al., 2015; Saini et al., 2021). Therefore, the I-TASSER (Iterative Threading Assembly Refinement) server was used to predict the entire structure of the Rv1250 protein. With the help of I-TASSER tool, five structures were obtained based on the C-Score (confidence score) (http://zhanglab.ccmb.med.umich.edu/I-TASSER/) (Yang and Zhang, 2015). The structure with the least value for C-Score was selected for further docking analysis. These selected tools for structure prediction are automatic homology modeling sever. There is no requirement of the input of a particular template as it automatically investigates a best fitting template of the target and presents results in minutes or hours. Energy minimization of selected template was done by using the SWISS-PDB (Protein Data Bank) Viewer (https://spdbv.vital-it.ch/) which is an automated server that offers an easily accessible interface allowing the investigation of numerous proteins at the same time (Arnold et al., 2006). The proteins can be overlaid in order to infer the structural alignments and relate their active sites or any other relevant parts (Jabeen et al., 2019). The model was validated by using Ramachandran Plot predicted from PROCHECK. Further, the validation of Homology model was done by using Verify-3D (http://servicesn.mbi.ucla.edu/Verify3D/), which ascertains the compatibility of an atomic model with its own amino acid sequence by allocating a structural class based on its environment and position (Luthy et al., 1992; Saini et al., 2019). Additionally, the model was validated by using ProSA integrated webserver (https://prosa.services.came.sbg.ac. at/prosa.php) to approximate deviation of Z-score from the high-resolution structures and to determine the error (Wiederstein and Sippl, 2007; Saini et al., 2021).

### 2.2 Active site prediction

The prediction of the active site was done by using Dogsite scorer which is an automated pocket detection tool, moreover it also analyze the drugability of the specific site by taking into consideration its geometric and physiochemical properties (http://dogsite.zbh.uni-hamburg.de) (Malik et al., 2019). The protein model was submitted in the software using this tool and the results were generated. Further validation of the results was done by Castp (http://sts-fw.bioengr.uic.edu/castp/calculation.php). Finally, it was modeled in PyMOL software, a cross-platform molecular graphic tool that has been extensively used for 3D visualization of surfaces, proteins, small molecules, nucleic acids, etc., (Wass et al., 2010; Yuan et al., 2017; Wu et al., 2018). PyMOL provides high-resolution images of macromolecules and it is easy to build a molecule from scratch (Yuan et al., 2017).

### 2.3 Ligand preparation

The three-Dimensional (3D) structure of the ligand isoniazid was retrieved from the PubChem database (http://www.pubchem.ncbi.nlm.nih.gov/) in a sdf format. This downloaded structure holds the information on the chemical properties and biological activities of small molecules. Further, sdf file was converted into pdb format by Open Babel and energy minimized by Universal Force Field (UFF) in PyRx 0.8 (O’Bayle et al., 2011; Dallankyan & Olson, 2015). The energy minimized isoniazid was considered for molecular docking against Rv1250.

### 2.4 Docking studies

Molecular docking is an extensively used computer simulation protocol to visualize the conformation of a receptor-ligand complex, where the receptor is usually a protein or a nucleic acid molecule and the ligand is either a small molecule or another protein. The docking of a specific ligand to the active site of a precise protein was done by using a server called SWISS-DOCK (http://www.swissdock.ch). There remains an automatic generation of the target protein structure as well as ligand structure for docking. Moreover, docking does not require computational inputs from users as all computations are made by server itself (Grosdidier et al., 2011). Finally, to validate the results of SWISS-DOCK, Autodock vina was used, which is one of the fastest and most widely used open-source programs for molecular docking (Singh et al., 2018; Dalal et al., 2019; Saini et al., 2019; Kesari et al., 2020). The grid box was generated by using Autodock tool and then the site-specific docking was done in the grid box. Hydrogen atoms and Kollman charges (4.03) were added to the protein, while Hydrogen atoms and Gasteiger charges were added on the ligand and both the files were converted in pdbqt format and saved. A potential grid box was generated using AutoGrid4 having spacing of 0.375Å (nearly one-fourth of the length of C-C covalent bond) (Table 1) (Kumari et al., 2021). The box dimensions were as 54 Å × 58 Å × 58Å and center point coordinates were as X = 83.911, Y = 69.166, Z = 77.416. In AutoDock Vina, total nine poses were generated by using the receptor and ligand files together with configuration file encompass grid box properties. An interaction of docking pose with active site residues was observed and the pose with higher binding affinity (−5.3 kcal/mol) was selected (Saini et al., 2019; Kumari et al., 2021).

**TABLE 1:** Grid box size and dimensions for molecular docking of isoniazid with Rv1250 in AutoDock tools.

Current total grid points/map	Number of points in X- dimension	Number of points in Y- dimension	Number of points in z -dimension	Spacing (angstrom)	X center grid box	Y center grid box	Z center grid box
191455	54	58	58	0.375	83.911	69.166	77.416

### 2.5 Molecular dynamic simulations

Molecular dynamics (MD) simulation is a technique that can be used effectively to understand macromolecular structure-to-function relationships (Ahmad & Kesavan, 2021). MD simulation is most widely used to evaluate the stability and to enhance low quality models (Sokkar et al., 2011). Here, it was used to observe the stability of a modelled structure, as well as the stable and static interaction between the ligand and the protein (Kesari et al., 2020; Singh et al., 2020; Saini et al., 2021; Kumari et al., 2022). For MD simulation, firstly GROMACS (Groningen Machine for Chemical Simulations) was used with the GROMOS96 43a1 force field for generating the topology and protein coordinates (Tran et al., 1996; Van Der Spoel et al., 2005). Then, the ligands were processed to create the topology and coordinates files by using PRODRG webserver (http://davapc1.bioch.dundee.ac.uk/cgi-bin/prodrg) (Kumari et al., 2020; 2021). The system was solvated in simple point charge (SPC) in a triclinic box (X = 10.71, Y = 8.73, and Z = 7.52 nm) having a minimum 1 nm distance between protein and edge of the box. Two distinct equilibration phases, i.e., constant number of particles, volume and temperature (NVT) and constant number of particles, pressure, and temperature (NPT) were run for 1 ns (Dalal et al., 2021), while final MD was run for 100 ns with a generation of coordinates at a regular interval of 10 ps. (Singh et al., 2022). The hydrogen bond occupancy and numbers between isoniazid-Rv1250 complex was generated by visual molecular dynamiucs (VMD) (Humphery et al., 1996; Swain et al., 2022).

## 3 Results and discussion

### 3.1 Prediction and validation of tertiary structure

**FIGURE 1:**
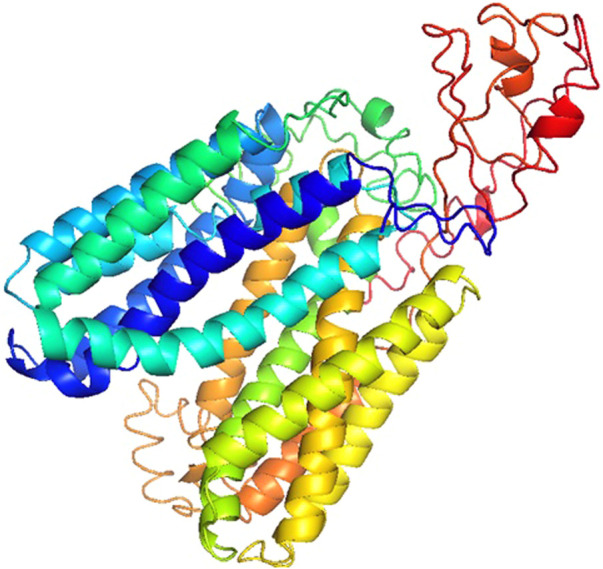
3DModel of Rv1250 from M.tuberculosis (α + β) with high C-score value build by I-TASSER.

The Rv1250 protein sequence was obtained from the NCBI. After the sequence retrieval, BLASTp (Basic Local Alignment Search Tool for Protein) was done for to check protein homologues of Rv1250 (Vyas et al., 2012). Among the homoglous structures, Crystal structure of a MFS transporter at 2.54 Å resolution (6G9X) showed a query coverage and identity of 23% and 28.19%, respectively with RV1250 (Binkowski et al., 2003). I-TASSER was used to generate five models of Rv1250 protein with varying C-score values, as shown in Table 2. The model with the highest C-score value, i.e., model-1 (c-score = −2.07) was selected for further performing the docking process (Figure 1).

**TABLE 2:** C-score of five models retrieved from I-TASSER for the selection of final model.

S.NO.	C-score
Model 1	−2.07
Model 2	−2.64
Model 3	−2.53
Model 4	−2.80
Model 5	−2.85

Model-1 was selected out of 5 models because it had the highest C-score value which signified that the model had a higher confidence (Armando et al., 2018).

This tool also predicted the normalized B-factor, which is a denomination that indicates the degree to which protein residues/atoms have inherent thermal mobility. This value is determined in I-TASSER, by threading the template proteins from the PDB along with the sequence profiles obtained from sequence databases. The reported B-factor profile corresponds to the target protein’s normalized B-factor, designated as B=(B′-u)/s, where B′ is the raw B-factor value, u is the mean and s is the standard deviation of the raw B-factors along the sequence. The resulted graph showed that coils had more mobility than the alpha-helix because the coils had a larger peak.

Ten templates for model building of Rv1250 protein were predicted (Table 3). However the template with a normalized Z-score >1 mean/least Z-score value was used by the I-TASSER for the alignment. The Z-score signifies the indicative of the quality of overall model and is utilized to verify that the input structure belongs to the range of scores usually located in the resident proteins of analogous size (Nawaz et al., 2014).

**TABLE 3:** 10 templates retrieved in building the final model by I-TASSER.

Rank	PDB hit	Iden1	Iden2	Cov	Norm. Z-Score
1	3wdoA	0.18	0.20	0.76	2.05
2	3wdoA	0.16	0.20	0.78	2.56
3	4zp0A	0.14	0.15	0.68	2.73
4	3wdoA	0.21	0.20	0.73	4.58
5	4zowA	0.13	0.15	0.67	2.37
6	3wdoA	0.16	0.20	0.75	8.24
7	1pw4	0.13	0.17	0.72	1.93
8	3wdoA	0.18	0.20	0.76	2.23
9	4zow	0.15	0.15	0.67	1.48
10	6e8j	0.18	0.21	0.71	2.20

After building the final protein model, it was prepared for the further docking process (Dar & Mir, 2017). The SWISS PDB viewer performed the energy minimization of the specific protein model (Supplementary Figure S3). In the predicted model, the elevated energies were eliminated using this technique, achieving local minima that were nearby to the native structure. Energy minimization is done as a definite step to resolve minute structural deformations, adverse interactions established during the modeling procedure (Waterhouse et al., 2018).

**FIGURE 2:**
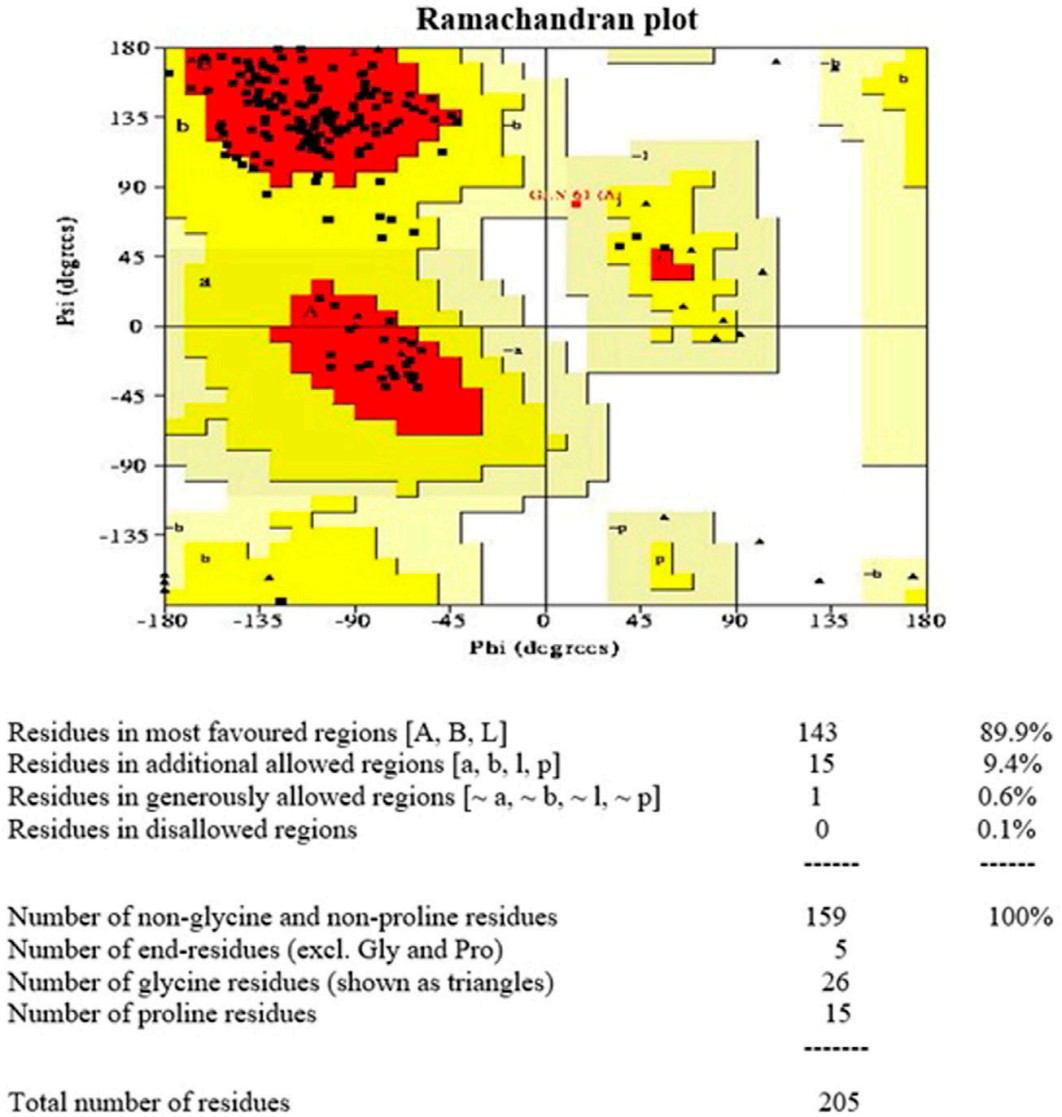
Ramachandran plot 3D model of Rv1250 generated using PROCHECK.

The Ramachandran plot of Rv1250 generated by PROCHECK showed that 89.9% of the amino acid residues are in the most favoured region, 9.4% in the additional allowed region, 0.6% in the generously allowed region and 0.1% in disallowed region as shown in Figure 2. The results showed that no residues were in the outliners region and similar scores were stated by Dhankhar et al. (2020), Dalal and Kumari (2022).

Furthermore, the 3D verification showed that the predicted model was valid for the further docking process of Rv1250 protein of *M. tuberculosis* (Abid et al., 2017). Following, results were concluded from the Verify-3D assessment model.

**FIGURE 3:**
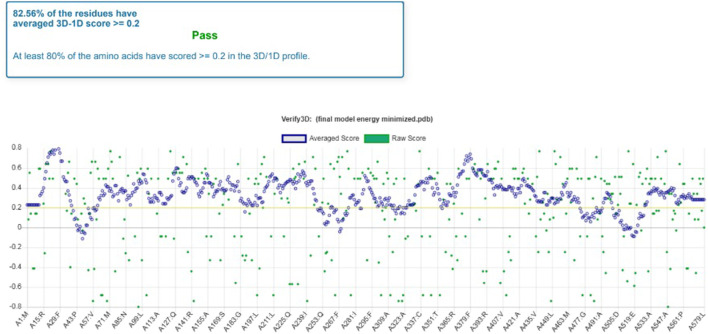
Model validation by Verify 3D which shows the percentage of average score of each residue.

• Residues (82.56%) had an average 3D-1D score ≥0.2
• Amino acids (about 80%) scored ≥0.2 in the 3D/1D profile (Figure 3).

**FIGURE 4:**
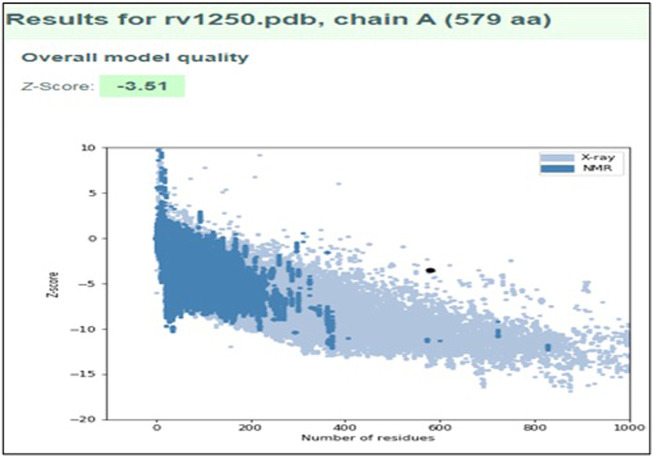
Model validation using ProSA plot representing the overall quality of the model and ProSA Z-score of modeled structure.

It was observed that the profile score of homology model was 82.56% which indicates that 82.56% of the amino acid residues had an average 3D-1D score of ≥0.2. Further, the model validation was done using ProSA and the z-score was obtained as −3.51 (Figure 4), which indicates that the modeled protein falls in the range of the X-ray elucidated protein structures (Kwofie et al., 2018; Saini et al., 2019; Kumari and Dalal, 2022). It has been also reported that a protein model is considered better with more negative z-score (Yakubu et al., 2017). Therefore, the results demonstrated that the model is reliable and can be considered for the molecular docking and dynamics (Panda et al., 2019).

### 3.2 Prediction of the active site

**FIGURE 5:**
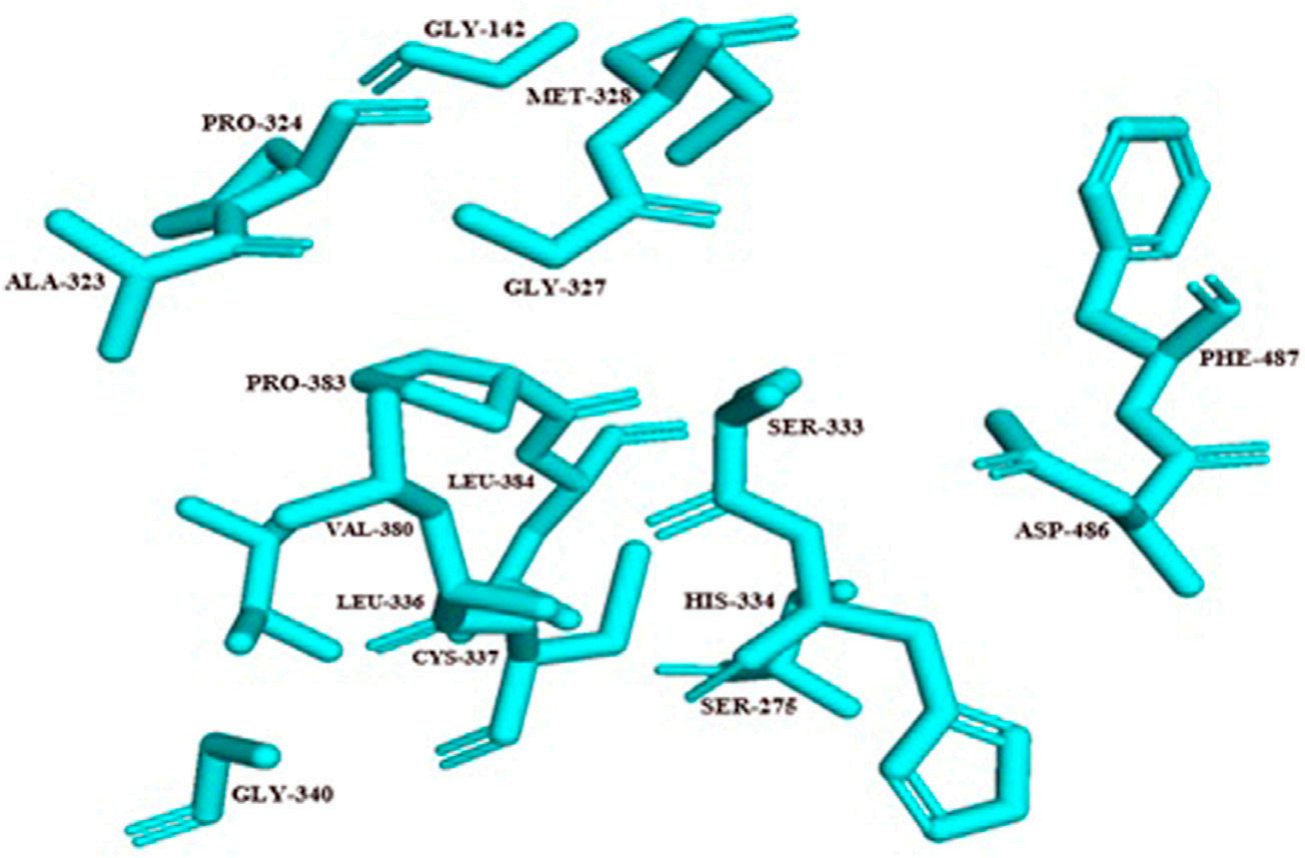
Active site residues of Rv1250 are represented in stick format in cyan color.

For the docking process, active site was predicted by the Dogsite scorer and Castp. These servers predicted an active site with sixteen amino acid residues [GLY142, SER275, ALA3232, PRO324, GLY327, MET328, SER333, HIS334, LEU336, CYS337, GLY340, VAL380, PRO383, LEU384, ASP486, PHE487] (Figure 5).

**FIGURE 6:**
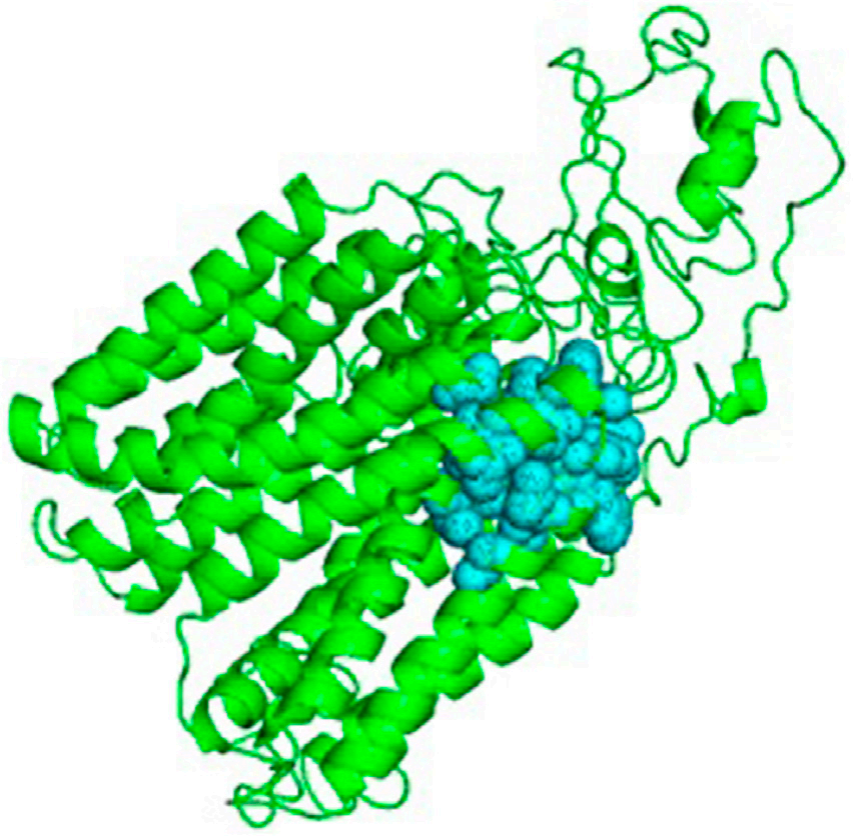
Cartoon representation of Rv1250 model (green color) along with predicted binding site in balls format (cyan color).

The 3D model with an active site is shown in Figure 6. Once the active site was identified, a suitable ligand, i.e., Isoniazid (molecular formula: C6H7N3O), for binding at this site was selected from the PubChem server, and basic properties and functions of isoniazid were also obtained from the same server. It is evident from the previous research that isoniazid is the best known and well-suited drug to such an infectious disease called TB (Egsmose et al., 1965). Over many decades, isoniazid is considered a highly selective and anti-tuberculosis therapeutic agent (Youatt, 1969; Zhang et al., 1996; Timmins & Deretic, 2006).

### 3.3 Molecular docking and visualization

Docking of small molecule compounds into a receptor binding site and evaluating the binding likeness of the composite is an imperative section of the structure-based drug design technique (Seeliger & de Groot, 2010). In this study, SWISS-DOCK and PyMOL were used to conduct the protein docking analysis (Dar & Mir, 2017). The protein model and the particular ligand were submitted to the server, and the docked model was received after validation by the SWISS-DOCK software. This software provides an automatic arrangement of protein-ligand structures, the diverse parameter sets and the expedient image and assessment of docking predictions which makes it a choice to be available to extensive viewers (Grosdidier et al., 2011).

**FIGURE 7:**
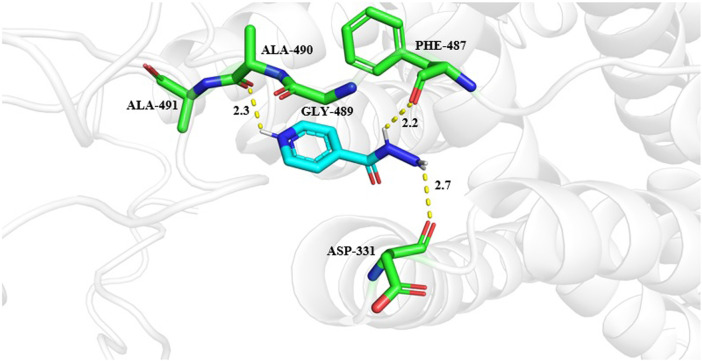
Interactions of isoniazid with Rv1250 predicted by molecualr docking. Interacting amino acid residues are shown in stick format in green color. While ligand is represented in cyan color. The hydrogen bonds between protein and ligands are shown in yellow dashed lines.

The molecular docking results were visualized by the PyMOL (Figure 7). Molecuolar docking performed by using AutoDock vina shows the binding affinity of −5.1 kcal/mol (Table 4). The ligand Isoniazid showed possible hydrogen bonding with ASP331, PHE487, GLY489, ALA490, and ALA491 residues, as displayed in Figure 7. The docking results showed that isoniazid (INH) was the most effective ligand to stop the action of *M. tuberculosis* by inhibiting the formation of mycolic acid (Takayama et al., 2005; Parulekar et al., 2013), which protects the bacterium from the antibiotic drug. Due to the efficiency of INH-NAD and INH-NAPD adducts to inhibit nucleic acid, and cell wall lipid synthesis, isoniazid provides an effective anti-tuberculosis blend with the efficiency of attacking multiple targets (Timmins & Deretic, 2006).

**TABLE 4:** Nine poses of the docked model with the binding affinities.

Mode	Affinity	Distance from best mode	
		rmsd l.b	rmsd u.b
1	−5.3	0.000	0.000
2	−5.2	1.788	2.419
3	−5.1	14.154	15.120
4	−4.9	16.499	18.252
5	−4.9	10.226	11.410
6	−4.8	1.737	2.171
7	−4.8	13.549	14.708
8	−4.8	21.928	22.680
9	−4.8	15.383	16.306

### 3.4 Molecular dynamic simulation

A molecular dynamic simulation is the tool used to assess protein-ligand system at the atomistic level and articulate on the stability of the protein-ligand complex in the dynamic environment. In the current study, different parameters, for instance, Root Mean Square Deviation (RMSD), Root Mean Square Fluctuation (RMSF), Radius of Gyration (Rg), Solvent Accessible Surface (SASA), and formation of hydrogen bonds were analyzed during the molecular simulation (Table 5).

**TABLE 5:** Average values of RMSD, RMFS, radius of gyration, SASA, intra protein hydrogen bond and inter molecular hydrogen bond of model protein, lidand and protein-ligand complex for the molecular simulation of 100 ns.

	RMSD	RMFS	Rg	SASA	Intra protein hydrogen bond	Inter molecular hydrogen bond
Model protein	0.621052	0.171169	2.476855	213.1524	398.1224	-
Protein-Ligand complex	0.532459	0.170058	2.449458	214.9703	390.4349	0.630537
Ligand	0.08702927	-	-	-	-	-

#### 3.4.1 Root Mean Square Deviation (RMSD)

**FIGURE 8:**
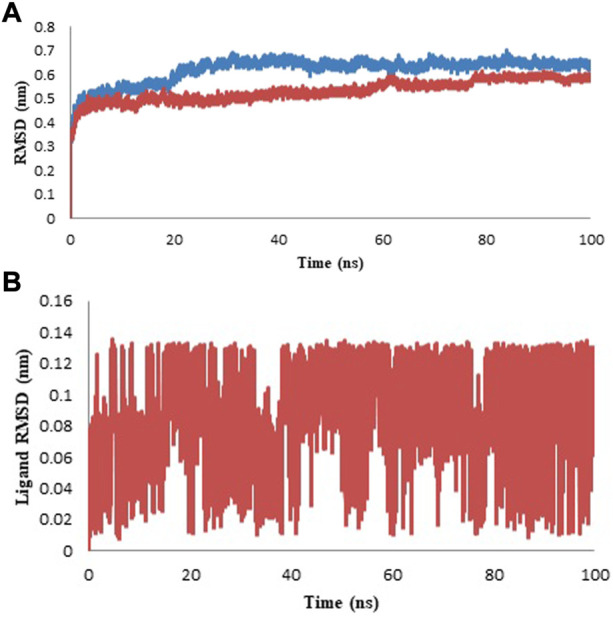
Protein and Ligand RMSD for the duration of 100 ns **(A)** Protein RMSD for model (blue color) and complex (red color). **(B)** Ligand RMSD of isoniazid is shown in red color.

The C-alpha backbone atoms of the model protein and protein-ligand complex were examined for the dynamic stability and conformational changes during the simulation by studying the RMSD. The developed RMSD plot revealed that the model protein and protein-ligand complex developed equilibrium at 25 ns and the system was observed to be stable up to 100 ns during the molecular dynamics simulation (Figure 8A). Ligand RMSD has been presented in Figure 8B and the average value of RMSD is given in Table 5. The RMSD study recommended that the ligand binding at the protein binding site was discovered to be stable and there was no effect on the steadiness of the C-alpha backbone of the protein.

#### 3.4.2 Root Mean Square Fluctuation (RMSF)

**FIGURE 9:**
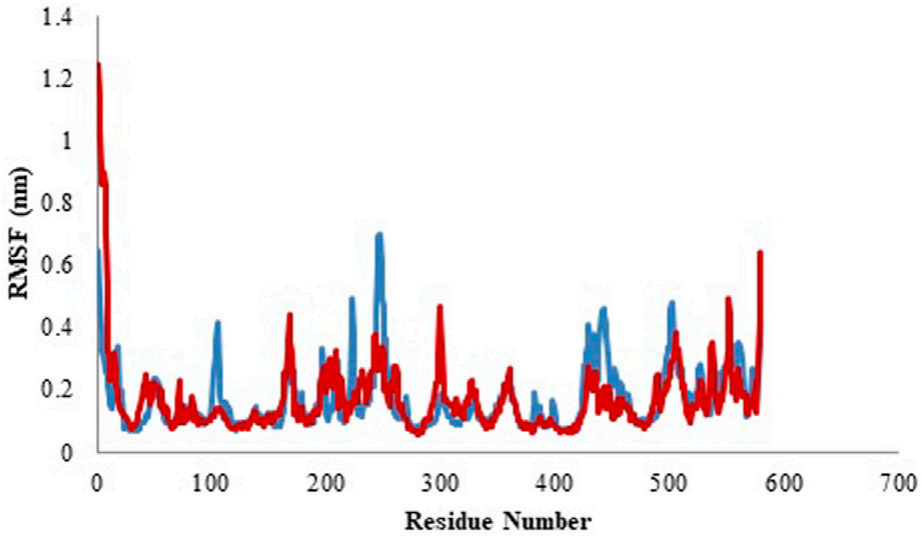
Root mean square fluctuation (RMSF) profile of model (blue color) and protein-ligand complex (red color).

RMSF of the sample structure was calculated to determine the tractability of the protein backbone. RFMS signifies the changeability of C-alpha coordinates from its usual location ll through the simulation. In general, the loosely structured loops are identified with reference to the high RMSF values, whereas, less flexibility is represented by the secondary elements in protein. Here, in current study, the residue mobility for model protein and protein-ligand complex was calculated. Subsequently, the graph was outlined against the number of amino acid residues corresponding to the MD simulation curve (Figure 9). Table 5 represents the average values of RMSF. The complete analysis reveals that the ligand molecule was fairly matched in the protein binding site and created a steady and durable protein-ligand complex (Saini et al., 2019).

#### 3.4.3 Radius of gyration (Rg)

**FIGURE 10:**
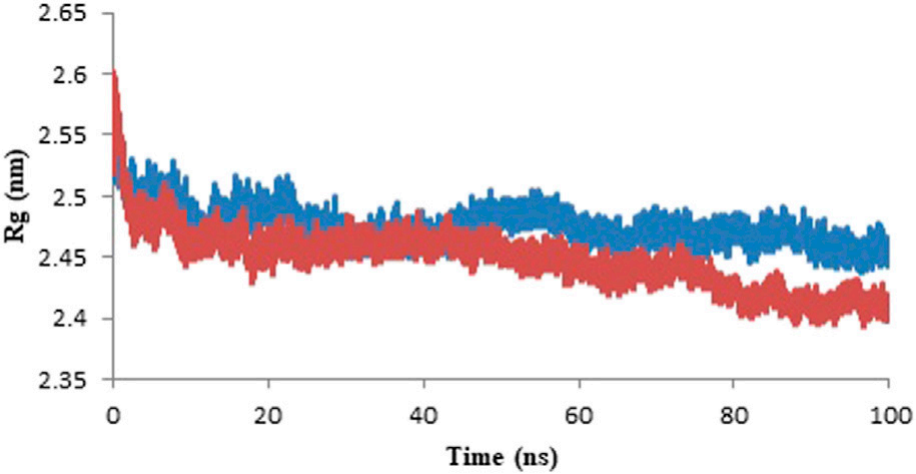
Radius of gyration of the model protein (blue color) and protein-ligand complex (red color) for the duration of 100 ns of molecular simulation.

Radius of gyration (Rg) displayed the total compactness of the protein structure during the simulation. It is calculated as the distance between the center of mass of all atoms of protein and its terminal in a particular time interval (Saini et al., 2019). The dynamic stability of a protein structure is determined by moderately less alteration in Rg which is further an indicative of a stable folded protein structure. The graph of the variation in Rg value against time represents that protein-ligand complex is more compactly packed as shown in Figure 10. The mean Rg of model protein and protein-ligand complex is given in Table 5 and the least alteration in Rg value is an indicative of compactly packed protein (Rathi, 2022).

#### 3.4.4 Solvent accessible surface area (SASA)

**FIGURE 11:**
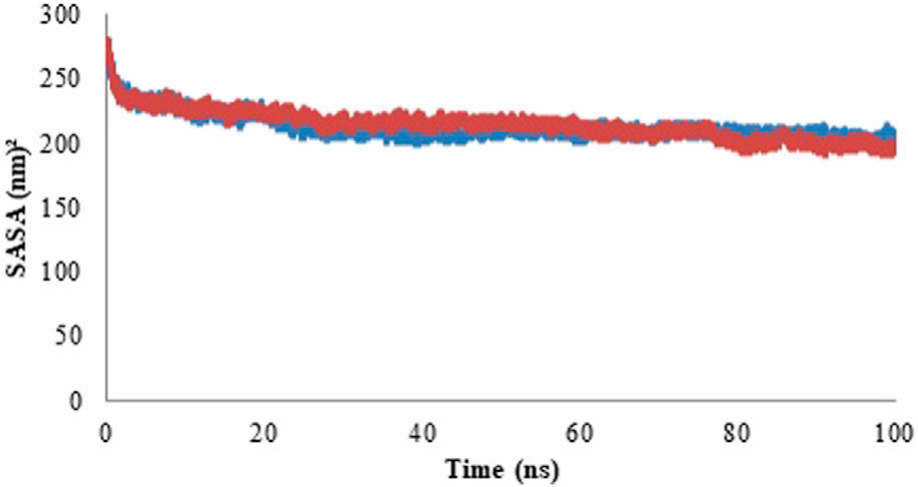
Solvent accessible surface area (SASA) profile of model protein (blue color) and protein-ligand complex (red color).

Solvent accessible surface area (SASA) determines the area of the solute which can interact with the solvent molecule via vander Waals forces (Saini et al., 2019). The SASA profile is represented in the form of graph in Figure 11 and the average values of SASA are given in Table 5. With an increase in the protein compactness, SASA of protein decreases, therefore, the alteration in a protein structure can easily be predicted with a variation in SASA. The result of SASA suggested the stable complex formation of the Rv1250 protein with isoniazid.

#### 3.4.5 Hydrogen bond analysis

**FIGURE 12:**
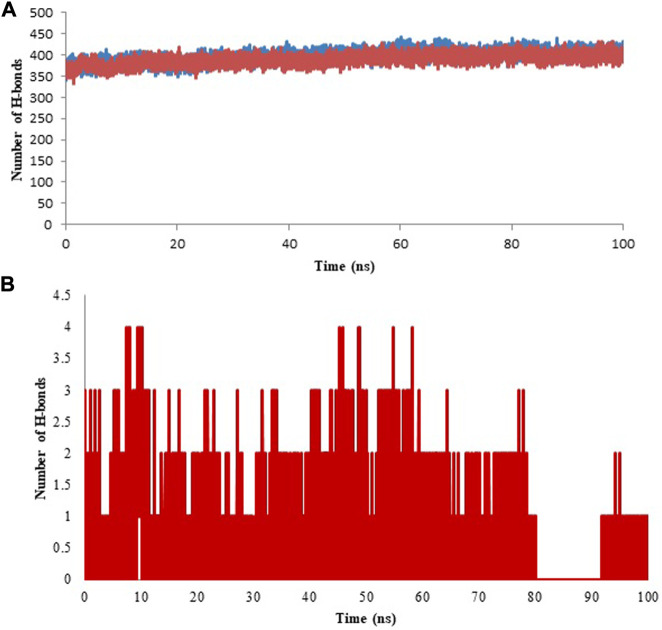
Hydrogen bond analysis for model and ligand bound complex analyzed for the time period of 100 ns of molecular simulation. **(A)** Intra hydrogen bond analysis for model and protein-ligand complex. **(B)** Inter molecular hydrgeon between isoniazid and Rv1250.

Hydrogen atom covalently attached to an electronegative atom can form hydrogen bonds within molecules or with other electro-negative atoms. Hydrogen bond of the GROMACS was utilized to discover the number and distribution of hydrogen bond in the model protein and the complex with an aim to investigate the stability of system through MD simulation period of 100 ns (Singh et al., 2022). Intra and Inter-molecular hydrogen bonding results of model protein and protein-ligand complex are given in Figure 12 (A & B, respectively) and the average values are displayed in Table 5. Further, hydrogen bond occupancy results showed that amino acid residues other than ASP331, PHE487, GLY489, ALA490, and ALA491 also contributed in the stabilization of isoniazid with Rv1250 (Table 6).

**TABLE 6:** The hydrogen bond occupancy of amino acid residues of Rv1250-isoniazid complex calculated from MD simulation of 100 ns.

Type of contribution	Amino acid residues (% of hydrgoen bond occupancies are in bracket)
Acceptor	ASP486 (36.32), PHE487 (22.09), MET328 (0.57), ASP331 (32.76), GLY489 (0.19), ALA490 (11.29), ILE329 (0.71), ALA535 (0.14), VAL492 (0.62), ILE330 (0.07), ALA537 (0.01), ALA536 (0.02), ARG332 (0.01), ALA533 (0.12), ALA534 (0.08), HIS334 (0.11), VAL485 (0.01), ALA491 (11.09), HIS139 (0.01), ALA493 (0.01), VAL326 (0.02), THR571 (0.33), THR488 (0.03), SER333 (8.16), LEU482 (0.01), SER577 (0.01), LEU579 (0.05), ASP205 (0.11), GLY208 (0.04), PHE207 (0.03), ASN230 (0.05), ALA229 (0.01), GLN227 (0.01), TRP206 (0.1), TRP231 (0.02), and ILE235 (0.01)
Donor	ALA490 (5.56), PHE487 (7.05), HIS139 (0.09), ARG390 (0.16), GLY489 (0.98), ALA535 (0.01), ASP331 (12.71), VAL492 (1.77), ALA536 (1.72), ARG539 (0.77), PHE325 (0.01), ALA491 (7.04), HIS334 (0.13), ARG332 (0.18), VAL209 (0.02), PHE207 (0.07), ASN230 (0.39), GLN227 (0.15), GLY210 (0.05), TRP206 (0.03), and TRP231 (0.01)

## 4 Conclusion

In order to fight tuberculosis, *M. tuberculosis* Rv1250 protein is a promising molecular target for the fabrication of new drugs. For this purpose, various bioinformatics tools were used to perform homology modeling and docking of this uncharacterized protein. The predicted model was energy minimized by SWISS PDB viewer. Further energy minimized model was validated by and Ramachandran plot, Verify-3D, and ProSA. Molecular docking results suggested that isoniazid interacts at the binding site and exhibits the non-covalent interactions with amino acid residues of RV1250. Further, molecular dynamics results displayed the stability of isoniazid with Rv1250.

## Data Availability

The original contributions presented in the study are included in the article/Supplementary Material, further inquiries can be directed to the corresponding authors.
