# Supplementary material for: Molecular modeling, simulation and docking of Rv1250 protein from Mycobacterium tuberculosis
Source: Front Bioinform. 2023 Apr 12;3:1125479. doi: 10.3389/fbinf.2023.1125479 (PMC10130521; doi:10.3389/fbinf.2023.1125479)
Supplement: Supplementary file 1 [file Table1.DOCX]

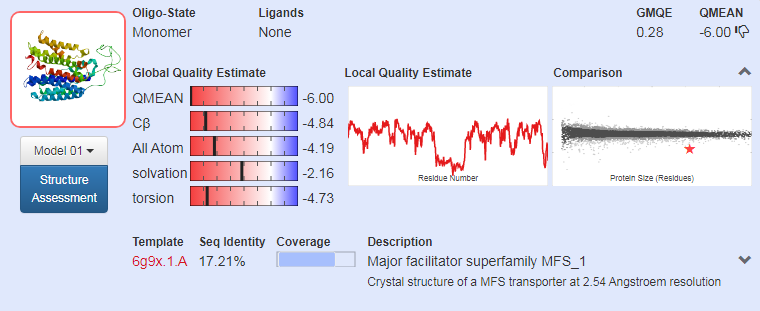


**Supplementary Figure 1:** Quality estimation by SWISS MODEL which stated that it was an unreliable model because of low sequence identity.

**
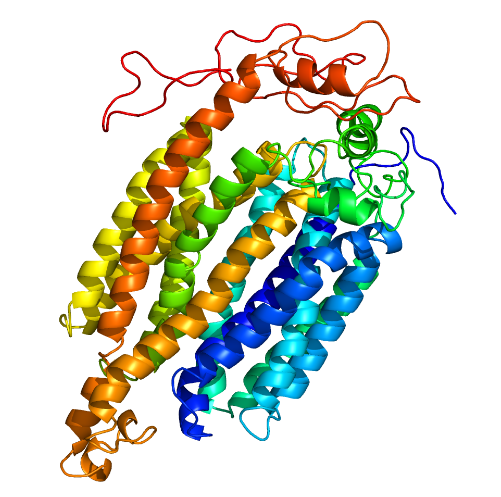
**

**Supplementary Figure 2:** 3D model building by PHYRE2 using the template 6G9X.


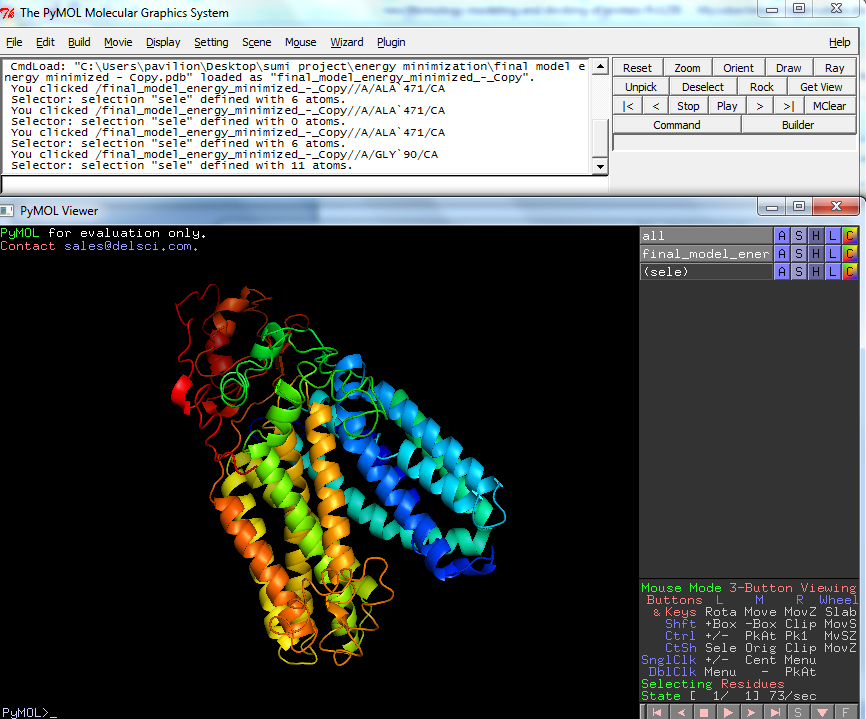


**Supplementary Figure 3:** Energy minimized model of protein represents the refinement of the model by the elimination of high energies from the protein.
